# Gestational trophoblastic neoplasia in the Western Brazilian Amazon: Patient characteristics and impact of referral center restructuring

**DOI:** 10.1016/j.gore.2026.102234

**Published:** 2026-10-19

**Authors:** Bruno Monção Paolino, Izildinha Maesta, Michelle Navarro Ferreira Paolino, Lia Mizobe Ono, Ross S. Berkowitz, Neil S. Horowitz

**Affiliations:** a Tocogynecology – CAPES-Dinter, São Paulo State University (UNESP), Medical School, Av. Professor Mário Rubens Guimarães Montenegro, s/n°, Botucatu, SP, 18618-687, Brazil; b State University of Amazonas (UEA), Av. Carvalho Leal, 1777 - Cachoeirinha, Manaus, AM, CEP 69065-001, Brazil; c Botucatu Trophoblastic Disease Center, São Paulo State University (UNESP), Medical School, Av. Professor Mário Rubens Guimarães Montenegro, s/n, Botucatu, SP, 18618-687, Brazil; d Amazonas Oncology Control Center (FCECON)- Rua Francisco Orellana, 215, Planalto Cep: 69040-010, Manaus, AM, Brazil; e New England Trophoblastic Disease Center, Division of Gynecologic Oncology, Department of Obstetrics, Gynecology and Reproductive Biology, Brigham and Women's Hospital, Dana Farber Cancer Institute, Harvard Medical School, 450 Brookline Avenue, Boston, MA, 02215, USA

**Keywords:** Gestational trophoblastic neoplasia, Referral center, Amazon, Treatment outcome

## Abstract

**Objective:**

To describe the demographic and clinical characteristics of patients with gestational trophoblastic neoplasia (GTN) managed at the Gestational Trophoblastic Disease Referral Center of Amazonas (RC-GTD Amazonas) and to assess the impact of the center's restructuring on treatment outcomes.

**Methods:**

This ambispective cohort study included women treated for GTN at RC-GTD Amazonas, AM, Brazil, between 2011 and 2022. Patients were grouped according to treatment period: before (2011–2019) and after (2020−2022) the center's restructuring. Demographic, clinical, and treatment data were collected from medical records. Outcomes included treatment characteristics, loss to follow-up, chemotherapy regimen changes, and mortality.

**Results:**

A total of 29 patients were treated, 11 before and 18 after restructuring. Most patients were young, of low socioeconomic status, and self-identified as *parda* or Indigenous. Post-molar GTN accounted for 76% of cases. After restructuring, there was a marked reduction in chemotherapy regimen changes and a significant decrease in loss to post-molar follow-up. Pre-treatment hCG levels were higher post-restructuring, likely reflecting more systematic and timely assessment. Overall mortality was 17%, with no significant difference between periods. All fatal cases were high-risk GTN and associated with advanced disease, treatment delay, and metastatic involvement. Hemorrhagic shock due to vaginal metastases was the leading cause of death.

**Conclusion:**

The restructuring of RC-GTD Amazonas improved treatment standardization and patient follow-up, even during the COVID-19 pandemic. Although mortality was unchanged, the findings highlight the importance of organized referral pathways, early diagnosis, and institutional preparedness to manage acute complications in geographically complex and resource-limited settings.

## Highlights

• First GTN cohort study from the Western Brazilian Amazon.
• Positive impact of reorganizing GTN care in a remote, underserved region.
• Center restructuring improved treatment standardization and follow-up.

## 1 Introduction

Gestational trophoblastic disease (GTD) is a rare pregnancy-related disorder classified into benign forms, including hydatidiform mole (HM) and placental site nodules, and malignant forms, collectively known as gestational trophoblastic neoplasia (GTN) (WHO Classification of Tumours Editorial Board, 2020, Lok et al., 2025). GTN comprises four subtypes: invasive mole (IM), choriocarcinoma, placental site trophoblastic tumor (PSTT), and epithelioid trophoblastic tumor (ETT). Although uncommon, GTD can lead to severe complications, with a risk of malignant transformation and high mortality if not properly treated. The International Federation of Gynecology and Obstetrics (FIGO) classifies GTN cases as low-risk (stage I–III, score 0–6) or high-risk (score ≥ 7) (FIGO Oncology Committee, 2002; Ngan et al., 2025).

The incidence of GTD varies worldwide, with higher rates in Asia and Latin America than in Europe and North America (Seckl et al., 2013; Horowitz et al., 2021). In Brazil, GTD occurs in approximately 1 in 200 to 1 in 800 pregnancies, depending on the region (Braga et al., 2014). However, no published studies have documented its prevalence in the state of Amazonas (AM).

The Western Brazilian Amazon comprises the states of Amazonas, Acre, Rondônia, and Roraima. The state of Amazonas is the largest in Brazil, covering a total area of 1,559,167.878 km^2^, with an estimated population of 4,321,616 in 2024 and a population density of 2.23 inhabitants/km^2^ (Instituto Brasileiro de Geografia e Estatística [Brazilian Institute of Geography and Statistics], 2024).

Advances in chemotherapy, human chorionic gonadotropin (hCG) assays, and the establishment of specialized referral centers have significantly improved GTN prognosis (Kohorn, 2014, Freitas et al., 2020). Studies indicate that patients treated at specialized centers achieve better outcomes than those not referred to specialized care (Dantas et al., 2012; Freitas et al., 2020). The most recent and largest national study conducted in Brazil reported a GTN mortality rate of 4.3% among 2186 cases treated in referral centers, highlighting the importance of centralized care (Braga et al., 2014). Nonetheless, many patients and healthcare providers remain unaware of the disease and the importance of early referral.

Before 2020, GTD management in Western Amazonia was decentralized, with patients receiving care from various healthcare providers, and no dedicated outpatient clinic for GTN existed. In response, the Gestational Trophoblastic Disease Referral Center of Amazonas (RC-GTD Amazonas) was established at the Amazonas Oncology Control Center (FCECON), a tertiary cancer hospital. This restructuring introduced standardized protocols based on national and international guidelines, facilitating timely referrals, case discussions, treatment, and patient follow-up.

Notably, this restructuring coincided with the COVID-19 pandemic (Buss et al., 2021) and severe logistical challenges, including record-breaking Amazonian river floods in 2021, which disrupted healthcare delivery. Moreover, in January 2021, a crisis caused by an oxygen shortage in the hospitals of Manaus (the state capital) underscored the region's healthcare vulnerabilities. Consequently, special strategies were implemented in response to these conditions.

This study aimed to provide an overview of the demographic and clinical characteristics of GTN patients managed at RC-GTD Amazonas from 2011 to 2022, and to assess the impact of RC-GTD Amazonas restructuring by comparing GTN treatment outcomes pre- and post-restructuring.

## 2 Methods

### 2.1 Study design and setting

This ambispective cohort study included women with GTN treated between 2011 and 2022 at RC-GTD Amazonas, located at FCECON, where all care is integrated into the Brazilian Public Health System (SUS), and totally free of charge. The study was divided into 2 periods. Data from first period (2011–2019)) were compiled retrospectively. The second period (2020–2022) included a prospective study conducted according to a protocol specially designed for this purpose. Patients with incomplete medical records were excluded.

Prior to 2020, when GTN care in the Amazonas region was decentralized, some patients received uterine evacuation in the private healthcare system, leading to potential underreporting of initial cases subsequently confirmed as GTN by histopathology.

In 2020, a new centralized model was implemented at RC-GTD Amazonas to standardize care and optimize treatment. This restructuring included the establishment of an integrated multidisciplinary team (gynecologists, clinical oncologists, social workers, and psychologists) following a standardized patient care flow. Additionally, national (Braga et al., 2018, Brasil. Ministério da Saúde. Secretaria de Atenção Primária à Saúde. Departamento de Ações Programáticas e Estratégicas, 2021) and international (Lok et al., 2020) guidelines were adopted to improve treatment standardization and efficiency.

### 2.2 Standardized care implementation

The restructured care model facilitated compliance with FIGO guidelines, improved referral efficiency, expedited case discussions and treatment initiation, enabled active patient follow-up and emotional support to the women affected.

Further improvements included:

• Ensuring continuous availability of quantitative hCG testing, previously irregular, at RC-GTD Amazonas.
• Offering injectable contraceptives at the follow-up clinic.
• Standardizing histopathological classification by requiring molar tissue block and slides for institutional review.
• Adopting FIGO staging guidelines, replacing chest CT with chest X-ray to minimize overtreatment.
• Establishing a partnership with Casa de Saúde Indígena (Casai) to facilitate access for indigenous patients across the Amazonas region.

### 2.3 Follow-up adaptations for remote patients

In Brazil, quantitative hCG is the gold standard for GTN follow-up; however, in many remote areas of Amazonas, this test is unavailable. To address this challenge, RC-GTD Amazonas implemented an alternative follow-up strategy inspired by the UK protocol. During the COVID-19 pandemic (2020), patients from remote areas without access to quantitative hCG were monitored using monthly qualitative hCG (urine or blood) testing after initial normalization. Results were transmitted via telemedicine (WhatsApp®). If a positive result occurred, patients were recalled for further evaluation. This approach considered the low risk (< 0.4%) of GTN recurrence after hCG normalization and the significant risks associated with long-distance travel by river.

This strategy proved effective during the second COVID-19 wave (2021) and the historic Amazon River flood of the same year, when travel restrictions were in place. Additional pandemic-related measures included:

• Telemedicine-based follow-up to minimize loss to follow-up.
• Biweekly hCG testing for patients with declining hCG curves.
• Early discharge of partial mole cases following three consecutive weekly negative hCG results.
• Delayed chemotherapy initiation for COVID-19-positive GTN patients.
• Cautious use of combination chemotherapy for high-risk cases, with intensive monitoring for neutropenia and ICU support when possible.
• Consideration of biweekly actinomycin D for low-risk GTN patients, though unfeasible due to medication unavailability.

### 2.4 Definition of GTN and data collection

GTN was defined according to the FIGO criteria (FIGO Oncology Committee, 2002) as a persistent hCG plateau for ≥3 weeks, hCG re-elevation for ≥2 weeks, or histologic diagnosis of choriocarcinoma.

Data regarding the pre-restructuring period (2011–2019) were collected retrospectively from physical and digital medical records following approval by the Research Ethics Committee (CEP) of FCECON (CAAE: 32779020.3.0000.0004). Data from the post-restructuring period (2020–2022) were prospectively recorded using a standardized study protocol, and patients provided informed consent as per IRB approval (CAAE: 16381619.3.0000.0004).

### 2.5 Statistical analysis

Statistical analyses were conducted using R software version 4.1.1 (2021-08-10). Data were presented in tables and graphs as medians, quartiles, and percentages. Continuous variables were assessed using the Shapiro-Wilk test for normality and the Levene test for homogeneity of variance.

Group comparisons were performed using ANOVA for normally distributed data, Kruskal-Wallis test for non-normal distributions, Pearson's chi-square test for categorical variables, and Mann-Whitney test for continuous numerical comparisons.

Statistical power was calculated based on Cohen's W and sample sizes, considering a significance level of 0.05. A power of 0.8 (80%) was set as the threshold to limit type II errors (β = 20%), ensuring result reproducibility. A *p*-value <0.05 was considered statistically significant.

## 3 Results

During the entire study period, 82 women with suspected gestational trophoblastic disease (GTD) were referred to RC-GTD Amazonas. Among them, 11 did not meet the diagnostic criteria for GTD or had incomplete medical records and were therefore excluded. Of the 71 confirmed GTD cases, 29 (41%) were diagnosed with GTN and included in this analysis. Of these, 11 were managed before the restructuring of the referral center (2011–2019) and 18 after the implementation of the new centralized model (2020–2022) (Fig. 1).

**Fig. 1:**
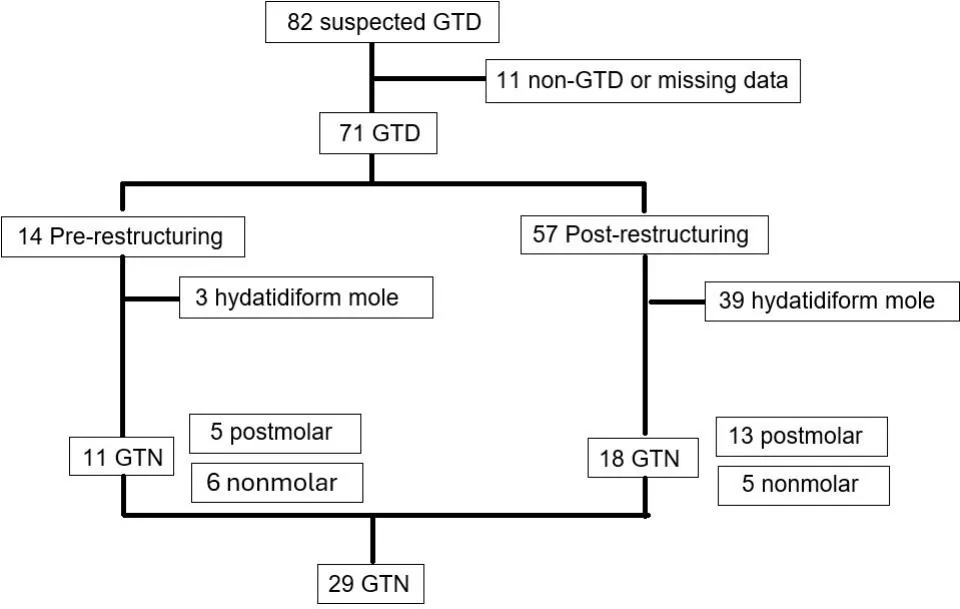
Patient flowchart.

### 3.1 Sociodemographic characteristics

Table 1 shows that there were no significant sociodemographic differences between patients treated before and after the restructuring of RC-GTD Amazonas. The median age at diagnosis was 32 years, and the median parity was approximately one child. Regarding self-reported race, 76% identified as *parda* (mixed race), 14% as Indigenous, 7% as White, and 3% as Black. The median educational level was 12 years, corresponding to completion of high school. The median distance from the patient's residence to the referral center was 18 km. In terms of marital status, 35% reported being in a stable union, 24% were single, 24% married, and 17% had no information recorded.

**Table 1:** Sociodemographic characteristics of patients with GTN treated at RC-GTD Amazonas, 2011–2022.

Variable	Overall	Pre-restructuring	Post-restructuring	p
	*N* = 29	(2011–2019)	(2020–2022)	
		*N* = 11	*N* = 18	
Age (median yrs., IQR)	32.0 (24.0; 39.0)	29.0 (25.5; 34.0)	35.0 (23.0; 45.0)	0.43^2^
Parity (median, IIQR)	1 (0; 2)	2.8 (1; 7)	1.3 (0; 6)	0.09^2^
Race				
Mixed (*Parda*)	22 (76%)	8 (73%)	14 (78%)	0.37^1^
Indigenous	4 (14%)	2 (18%)	2 (11%)	
White	2 (7%)	0 (0%)	2 (11%)	
Black	1 (3%)	1 (9%)	0 (0%)	
Education (median years, IQR)	12 (12; 16)	12 (6.5; 14,5)	12.5 (12.0; 15.7)	0.25^2^
Household income (BRL)(median, IQR)	–	–	1750 (821; 3500)	–
Prior knowledge of disease	–	–	1 (5%)	–
Distance to RC (median km, IQR)	18.0 (10.8; 369)	80 (8.5; 468)	17.5 (12; 321)	1.00^2^
Marital status				
Stable union (n, %)	10 (35%)	1 (9%)	9 (50%)	0.08^1^
Married (n, %)	7 (24%)	1 (9%)	6 (33%)	
Single (n, %)	7 (24%)	4 (36%)	3 (17%)	
Unknown (n, %)	5 (17%)	5 (46%)	0 (0%)	
Planned pregnancy (n, %)	–	–	3 (17%)	–

IQR- interquartile ranges (q1 = 25%, q3 = 75%), n- total number of patients, (%) percentage. **Abbreviations:** GTN – Gestational Trophoblastic Neoplasia; GTD – Gestational Trophoblastic Disease; RC – Referral Center; km – kilometers.

1 *p* < 0.05 – Chi-square test.

2 *p* < 0.05 – Mann–Whitney test.

### 3.2 Clinical characteristics and treatment

As shown in Table 2**,** most cases (76%) were post-molar GTN, while 24% were non-molar. Among the 22 post-molar cases, seven (24%) were choriocarcinoma, and four (13%) had no available histopathological analysis. The median interval between uterine evacuation and initiation of chemotherapy was 5 months in the pre-restructuring period and 3 months after restructuring (*p* = 0.10). Median pre-treatment hCG level was 88,938 IU/L (IQR 9083–246,767).

**Table 2:** Clinical characteristics and treatment of patients with GTN treated at RC-GTD Amazonas, 2011–2022.

Variable	Overall	Pre-restructuring	Post-restructuring	p
	*N* = 29	(2011–2019) (*n* = 11)	(2020–2022) (*n* = 18)	
GTN Type				
GTN post-molar (n, %)	22 (76)	7 (64)	15 (83)	0.22^1^
GTN non-molar (n, %)	7 (24)	4 (36)	3 (17)	
UE/chemo interval (mos.)	4 (3–5,5)	5 (3–7)	3 (2–5)	0.10^2^
Pretreatment hCG (IU/L)	88.938	15.000	105.499	
(median, IQR)	(9.083; 246.767)	(6.332; 41.447)	(10.000; 387.068)	
Metastasis (n, %)	17 (58)	8 (72)	9 (50)	0.22^1^
Metastatic site^(⁎)^				
Lung (n, %)	12 (53)	6 (67)	6 (42)	
Vagina (n, %)	4 (17)	1 (11)	3 (21)	
Pelvis (n, %)	4 (17)	2 (22)	2 (15)	0.48^1^
Urinary tract (n, %)	2 (9)	0 (0)	2 (15)	
Liver (n, %)	1 (4)	0 (0)	1 (7)	
FIGO score				
Low risk (n, %)	21 (72)	7 (64)	14 (78)	0.30^1^
High risk (n, %)	8 (28)	4 (36)	4 (22)	
Chemotherapy ^(⁎⁎)^				
Single agent (n, %)	16 (60)	6 (55)	10 (62)	0.82^1^
Multiple agents (n, %)	11 (40)	5 (45)	6 (38)	
Chemo regimen switch ^(⁎⁎)(n, %)^	6 (22)	5 (45)	1 (6)	0.01^1^
Reason for switch ^(⁎⁎⁎)^				
Treatment failure (n, %)	4 (60)	3 (60)	1 (100)	
G4 Mucositis (n, %)	1 (20)	1 (20)	0 (0)	0.43^1^
Allergy (n, %)	1 (20)	1 (20)	0 (0)	
Chemo adverse events (n, %)				
Mucositis (n, %)	8 (34)	–	8 (34)	
Nausea/Vomit (n, %)	7 (29)	–	7 (29)	–
Alopecia (n, %)	5 (21)	–	5 (21)	
Anemia (n, %)	2 (8)	–	2 (8)	
Other (n, %)	2 (8)	–	2 (8)	
Hysterectomy (n, %)	7 (24)	4 (36)	3 (16)	0.22^1^
TAH Indication ^(⁎⁎⁎⁎)^(n, %)				
Transvaginal bleeding (n, %)	4 (58)	3 (75)	1 (33)	
First-line treatment (n, %)	2 (28)	1 (25)	1 (33)	0.43^1^
Residual disease (n, %)	1 (14)	0 (0)	1 (33)	
Lost to follow-up (n, %)	2 (7)	2 (18)	0 (0)	0.01^1^
Death (n, %)	5 (17)	3 (27)	2 (12)	0.26^1^

Values are presented as medians and interquartile ranges (q1 = 25%, q3 = 75%), total number of patients (n), or percentage of patients (%).

**Abbreviations::** GTN – Gestational Trophoblastic Neoplasia; GTD – Gestational Trophoblastic Disease; RC – Referral Center; U.E/chemo interval- interval between uterine evacuation and chemotherapy initiation; hCG – human chorionic gonadotropin; IU/L – international units per liter; TAH – Hysterectomy.

1 *p* < 0.05 – Chi-square test.

2 *p* < 0.05 – Mann–Whitney test.

According to FIGO scoring, 72% of cases were classified as low-risk and 28% as high-risk GTN. Metastatic disease was present in 58% of all patients, most commonly involving the lungs (53%), followed by the vagina (17%) and pelvis (17%). Notably, the frequency of metastasis was 72% pre-restructuring and 50% after restructuring (*p* = 0.22).

Single-agent chemotherapy was the most frequent first-line treatment (60%), whereas 40% received multi-agent regimens. A significant reduction in chemotherapy regimen changes was observed after the restructuring (6% vs. 45%, *p* = 0.01). Among the six patients who required a change of regimen, the main reasons were treatment failure (60%), grade 4 mucositis (20%), and allergy (20%).

Adverse events occurred in 34% of patients, mainly mucositis (34%) and nausea or vomiting (29%). However, data on chemotherapy-related adverse events were collected only during the prospective phase due to lack of documentation in retrospective records.

Hysterectomy was performed in 24% of cases, primarily due to vaginal bleeding (58%) or as a first-line therapeutic option (28%). Loss to follow-up significantly decreased after the restructuring (0% vs. 18%, *p* = 0.01).

Overall mortality was 17% (5/29). Although not statistically significant, mortality was lower in the post-restructuring period (12% vs. 27%, *p* = 0.26).

### 3.3 Comparison between survivors and non-survivors

Table 3 shows that five patients (17%) died during the study period, while 24 (83%) achieved survival. Non-survivors were slightly older than survivors (median 34 years vs. 30.5 years). In addition, all were indigenous or *parda* and presented with vaginal bleeding at GTN diagnosis (100%, *p* = 0.04).

**Table 3:** Comparison of sociodemographic and clinical characteristics between survivors and non-survivors with GTN at RC-GTD Amazonas (2011−2022).^1^, ^2^

Variable	Overall (***n*** = 29)	Death (***n*** = 5)	Survival (***n*** = 24)	p
Age (yrs, median, IQR)	32 (24; 39)	34 (29; 39)	30.5 (23; 37)	0.43^2^
Race				
Mixed (*Parda*) (n, %)	22 (76)	3 (60)	19 (79)	0.28^1^
Indigenous (n, %)	4 (14)	2 (40)	2 (8)	
White (n, %)	2 (7)	0 (0)	2 (8)	
Black (n, %)	1 (3)	0 (0))	1 (5)	
Education (yrs, median, IQR)	12 (12–16)	12 (3–16)	12 (12–15)	0.61^2^
Provenance (n, %)				
Manaus (Capital city) (n, %)	15 (52))	2 (40)	13 (55)	0.56^1^
Outside the capital city (n, %)	14 (48)	3 (60)	11 (45)	
Distance to the RC(median km. IQR)	18 (10.8; 369)	369 (10; 382)	17.5 (10; 287)	0.48^2^
Clinical condition				
Transvaginal bleeding (n, %)	19 (65)	5 (100)	14 (58)	
Hyperemesis (n, %)	3 (10)	0 (0)	3 (13)	0.04^1^
Asymptomatic (n, %)	3 (10)	0 (0)	3 (13)	
Other (n, %)	4 (15)	0 (0)	4 (16)	
GTN type				
Postmolar GTN (n, %)	22 (76)	3 (60)	19 (79)	0.36
Nonmolar GTN (n, %)	7 (24)	2 (40)	5 (21)	
Mean antecedent pregnancy/ chemotherapy (n, %) initiation interval (mos.)	4,6 (1−11)	6,2 (3−11)	4,3 (1–11)	0.04^2^
Pretreatment hCG (IU/L)	293.534	57.680		
	(9.083–246.767)	(246.767–340.301)	(9.083–194.231)	0.20^2^
Metastases (n, %)	17 (58)	5 (100)	12 (50)	0.03^1^
Metastatic site^(⁎)^(n, %)				
Lung (n, %)	11 (58)	5 (62)	6 (54)	0.04^1^
Vagina (n, %)	4 (21)	3 (38)	1 (10)	
Other (n, %)	4 (21)	0 (0)	4 (36)	
FIGO score (n, %)				
Low-risk (n, %)	8 (27)	0 (0)	8 (33)	0.01^1^
High-risk (n, %)	21(72)	5 (100)	16 (67)	
Chemotherapy (n, %)				
Single-agent (n, %)	16 (55)	1 (20)	16 (64)	0.08^1^
Multiagent (n, %)	11 (37	4 (80)	7 (29)	
Hysterectomy (n, %)				
Yes	7 (25)	1 (20)	6 (25)	0.81^(1)^

Values are presented as medians and interquartile ranges (q1 = 25%, q3 = 75%), total number of patients (n), or percentage of patients (%).

**Abbreviations:** GTN – Gestational Trophoblastic Neoplasia; RC – Referral Center; GTD – Gestational Trophoblastic Disease; km – kilometers; hCG – human chorionic gonadotropin.

1 *p* < 0.05 – Chi-square test.

2 *p* < 0.05 – Mann–Whitney test.

The median interval between uterine evacuation and initiation of chemotherapy was significantly longer among fatal cases (6.2 months vs. 4.3 months, *p* = 0.04). All five deaths occurred in patients with metastatic disease (100% vs. 50%, *p* = 0.03). Three patients had hemorrhage due to vaginal metastasis, required massive transfusions (∼10 packed red blood cells) and died before chemotherapy was started. The other two died during treatment: one from respiratory failure caused by multiple lung metastases after 10 EMA/CO cycles, and the other from methotrexate-induced epidermal necrosis after 2 EMA/CO cycles. According to FIGO scoring, all non-survivors were classified as high-risk GTN (*p* = 0.01).

## 4 Discussion

This study provides the first description of patients with gestational trophoblastic neoplasia managed in the state of Amazonas and evaluates the impact of restructuring a regional referral center in a geographically complex and underserved setting. The cohort consisted predominantly of young women who self-identified as *parda* or Indigenous and reported low household income. Many pregnancies were unplanned, and only a minority of women had prior knowledge of trophoblastic disease. These sociodemographic characteristics highlight the social vulnerability of the population served by the referral center and underscore the particular challenges faced by Indigenous women in the region.

Although the median distance between patients' residences and the referral center appeared relatively short, distance alone is an inadequate indicator of healthcare accessibility in the Amazon. Many municipalities lack road connections to Manaus, and transportation often depends on small river vessels navigating extensive and challenging waterways. These logistical barriers may limit access to specialized care for women living in remote communities, suggesting that the cohort may not fully reflect the broader population at risk for GTN in the Western Brazilian Amazon.

When assessing the impact of the referral center restructuring, treatment indicators did not worsen during the COVID-19 pandemic compared with the historical cohort. Rather, improvements were observed in key domains after 2020. There was a marked reduction in the need for chemotherapy regimen changes, likely related to treatment standardization based on FIGO risk scoring, as well as a significant decrease in loss to post-molar follow-up. This latter improvement coincided with the implementation of new active patient-tracking strategies, including telemedicine-based monitoring, which facilitated follow-up in a geographically challenging setting (de Assis et al., 2022). Although mortality did not decrease significantly, these findings are consistent with the expected benefits of centralized, protocol-based care (Kohorn, 2014, Freitas et al., 2020) and suggest that the reorganization of RC-GTD Amazonas contributed to more standardized treatment pathways and improved continuity of care.

Notably, pre-treatment hCG levels were higher in the post-restructuring period compared with before. This difference should be interpreted in the context of changes in clinical practice. Before 2020, hCG measurements were often performed irregularly, and in many cases treatment was initiated without an updated quantitative hCG value obtained within 24 h, relying instead on clinical presentation (Lok et al., 2025). After the restructuring, systematic and timely hCG assessment became routine, likely resulting in more accurate documentation of pre-treatment hCG levels.

Significant differences also emerged between survivors and non-survivors. Fatal cases were all classified as high-risk GTN, had a longer interval between the antecedent pregnancy and the initiation of chemotherapy, and presented with a higher frequency and number of metastatic sites. The shorter treatment delay observed after restructuring supports the hypothesis that diagnostic and referral pathways became more efficient in recent years. These patterns align with established prognostic factors described in Brazilian and international cohorts, in which high-risk disease, metastatic involvement, and delayed treatment initiation are consistently associated with poorer outcomes (Maestá et al., 2020, Kohorn, 2014).

Among the five deaths recorded, hemorrhagic shock was the cause in three cases, all associated with bleeding vaginal metastases. Vaginal bleeding therefore remains a predominant cause of mortality associated with GTN in Amazonas. These findings highlight the importance of early diagnosis, timely initiation of chemotherapy, and adequate institutional preparedness to manage hemorrhagic complications (Parker et al., 2025). Previous studies from specialized referral centers have shown that early mortality in GTN is frequently associated with advanced disease at presentation, high tumor burden, and acute complications such as hemorrhage, rather than lack of chemosensitivity (Alifrangis et al., 2013; Neubauer et al., 2015). In this context, effective management depends not only on appropriate chemotherapy regimens but also on the availability of experienced multidisciplinary teams capable of delivering rapid, coordinated care in the acute setting (Chan Wah Hak et al., 2019). This is particularly relevant in regions such as the setting studied here, where delays in diagnosis and barriers to access may contribute to more advanced and unstable clinical presentations.

The main limitations of this study include the small sample size and the retrospective design of part of the cohort, which may have resulted in incomplete documentation of some clinical variables. Additionally, the cohort may not fully represent women living in the most remote areas of the Western Brazilian Amazon, where access to specialized care is particularly challenging. Therefore, underdiagnosis of GTN in parts of the region cannot be excluded. Despite these limitations, this study provides the first region-specific description of GTN in the Western Brazilian Amazon and offers valuable insight into patient management in a geographically complex and resource-limited setting. The presence of pre- and post-restructuring cohorts also enabled an initial evaluation of organizational changes implemented at RC-GTD Amazonas.

## 5 Conclusion

This study demonstrates measurable improvements in treatment standardization and patient follow-up after the restructuring of RC-GTD Amazonas. Although mortality remained unchanged, the findings highlight the importance of organized referral pathways and timely initiation of therapy in regions with substantial logistical challenges. These results may help guide future efforts to strengthen GTD care in the Western Brazilian Amazon and support similar initiatives in other underserved settings.

## Declaration of generative AI and AI-assisted technologies in the manuscript preparation process

During the preparation of this work the author(s) used ChatGPT (OpenAI) to assist with language refinement and improvement of readability. After using this tool/service, the author(s) reviewed and edited the content as needed and take(s) full responsibility for the content of the published article.

## Data statement

Due to ethical concerns, the supporting data cannot be made openly available. De-identified data may be made available to qualified researchers upon reasonable request and with approval from the institutional IRB.

## CRediT authorship contribution statement

**Bruno Monção Paolino:** Writing – original draft, Data curation, Conceptualization. **Izildinha Maesta:** Validation, Supervision, Funding acquisition. **Michelle Navarro Ferreira Paolino:** Investigation. **Lia Mizobe Ono:** Resources, Investigation. **Ross S. Berkowitz:** Writing – review & editing. **Neil S. Horowitz:** Writing – review & editing.

## Ethics declaration

Written informed consent to take part in the study and to publish the article has been obtained from all participants or their legal representatives. The privacy rights of participants have been observed.

This study was performed in compliance with relevant laws, regulatory frameworks and guidelines where the research took place. This study was approved by the Research Ethics Committee (CEP) of Fundação Centro de Controle de Oncologia do Estado do Amazonas (FCECON). (Approval No. CAAE: 16381619.3.0000.0004).

## Funding

This study was financed in part by the Coordenação de Aperfeiçoamento de Pessoal de Nível Superior – Brasil (CAPES) - Finance Code 001, PROEX – AUXPE n° 3155/2025. The funding agencies had no direct role in the generation of the data or the manuscript.

## Declaration of competing interest

The authors declare that they have no known competing financial interests or personal relationships that could have appeared to influence the work reported in this paper.
